# Microbial dysbiosis drives colorectal carcinogenesis via integrated inflammatory, metabolic, and biofilm pathways

**DOI:** 10.3389/fmicb.2026.1795882

**Published:** 2026-06-02

**Authors:** Asma Bachir, Alaa Muayad Altaie, Riyad Bendardaf, Iman M. Talaat, Rifat Hamoudi

**Affiliations:** 1 Research Institute of Medical and Health Sciences, University of Sharjah, Sharjah, United Arab Emirates; 2 Clinical Sciences Department, College of Medicine, University of Sharjah, Sharjah, United Arab Emirates; 3 Center of Excellence for Precision Medicine, Research Institute of Medical and Health Sciences, University of Sharjah, Sharjah, United Arab Emirates; 4 Oncology Unit, University Hospital Sharjah, Sharjah, United Arab Emirates; 5 Pathology Department, Faculty of Medicine, Alexandria University, Alexandria, Egypt; 6 BIMAI-Lab, Biomedically Informed Artificial Intelligence Laboratory, University of Sharjah, Sharjah, United Arab Emirates; 7 Division of Surgery and Interventional Science, University College London, London, United Kingdom; 8 ASPIRE Precision Medicine Research Institute Abu Dhabi, University of Sharjah, Sharjah, United Arab Emirates

**Keywords:** colorectal cancer, gut microbiome, inflammatory signaling, microbial dysbiosis, NF-κB/STAT3 activation

## Abstract

Colorectal cancer (CRC) arises from a multifaceted interplay among the intestinal microbiota, chronic inflammation, and host genomic instability, with microbial dysbiosis serving as an active driver rather than a by-product of malignant transformation. Genotoxic *Escherichia coli* (colibactin-positive), enterotoxigenic *Bacteroides fragilis*, and *Fusobacterium nucleatum* contribute to distinct stages of CRC progression by engaging the DNA-damage response and activating β-catenin–dependent Wnt signaling and NF-κB/STAT3 transcriptional programs controlling pro-inflammatory (*IL-6, IL-8*), pro-survival (*BCL-2, BCL-XL*), and proliferative (*MYC, CCND1*) gene expression.. Here, we propose a tri-axial pathogenic framework in which (i) cyclic dinucleotide–mediated activation of the cGAS–STING pathway engages TBK1–IRF3 and NF-κB signaling, driving type I interferons (*IFN-*β) and pro-inflammatory cytokines (*IL-6, TNF-*α) that couple microbial genotoxic stress to innate inflammation; (ii) altered microbial metabolites, including indoles and bile acids, reprogram AhR and FXR/TGR5 signaling; and (iii) crypt-anchored biofilms spatially amplify *IL-6* leading to activation of STAT3, epigenetic silencing of tumor suppressors, and immune evasion. This review critically synthesizes current evidence supporting these axes and maps them onto CRC molecular subsets and tumor location. Recognition of these integrated microbial-host circuits identifies mechanistically grounded candidates for biomarker development, microbiome-based diagnostics, and targeted interventions to restore microbial and immune equilibrium, thereby providing a refined framework for the molecular classification and precision management of CRC.

## 1 Introduction

Colorectalcancer (CRC) is the third most common cancer worldwide, with incidence continuing to rise despite advances in early detection and treatment (Akimoto et al., 2021). The etiology of CRC is complex, involving an interplay among genetic predispositions, environmental factors, and, more recently, disruption of the gut microbiome (Cintoni et al., 2025). The human gastrointestinal tract is colonized by trillions of microorganisms, including bacteria, archaea, viruses, and fungi. This microbiome plays a crucial role in maintaining host health by aiding digestion, regulating the immune system, and protecting against pathogenic organisms (Thursby and Juge, 2017). However, dysbiosis, or an imbalance in the gut microbiota, has increasingly been recognized as a contributing factor in the development of various diseases, including CRC (Singh et al., 2025). The incidence of CRC among individuals under 50 has nearly doubled in recent years. This alarming trend is accompanied by the presentation of more aggressive forms of the disease in younger populations, often diagnosed at advanced stages when treatment is less effective. As a result, survival rates among younger CRC patients are significantly poorer than those among older patients (Cavestro et al., 2018). Experts have attributed this rise to a combination of factors, including shifts in lifestyle and dietary habits, such as increased consumption of processed foods and red meats, obesity, and sedentary behaviors. Furthermore, disruptions in the gut microbiome and chronic inflammation have increasingly been implicated as contributing factors in early-onset CRC (Saha et al., 2024). Unlike traditional CRC cases in older patients, younger patients often lack access to early screening protocols and may dismiss symptoms as benign, leading to delayed diagnoses. Many young CRC patients report initial symptoms such as abdominal pain, rectal bleeding, or changes in bowel habits, which are often misdiagnosed or overlooked. These findings highlight the critical need for heightened awareness among healthcare providers and the public about the rising trend of CRC in younger individuals. Early detection through expanded screening guidelines, public education on symptom awareness, and research into the unique biological and environmental factors driving this trend are essential to improving survival outcomes. This growing burden of CRC in younger populations calls for a proactive approach to prevention, early diagnosis, and treatment tailored to this demographic (Cho, 2025).

The increasing consumption of junk food, characterized by high quantities of refined sugars, unhealthy fats, artificial additives, and minimal dietary fiber, has been strongly correlated with the rising global incidence of CRC. This dietary shift toward high-volume intake of ultra-processed foods exacerbates disruptions in gut microbiota composition, driving a dysbiotic state that amplifies the risk of CRC. This dysbiosis is characterized by the overgrowth of certain pathogenic bacteria, such as *Fusobacterium nucleatum* and *Bacteroides fragilis*, while diminishing protective species, such as *Lactobacillus* and *Bifidobacterium*. This microbial shift weakens the intestinal barrier, stimulates chronic inflammation, and dysregulates immune responses, creating a microenvironment favorable to tumor initiation and progression (Wang et al., 2012). Furthermore, the high-temperature cooking methods commonly used in junk food preparation produce elevated levels of carcinogenic compounds such as heterocyclic amines (HCAs), polycyclic aromatic hydrocarbons (PAHs), and acrylamide. These compounds cause DNA damage by forming DNA adducts, which can result in mutations if the damage is not properly repaired. Simultaneously, the dysbiotic gut microbiome produces reactive oxygen species (ROS) and inflammatory mediators, further exacerbating DNA damage and promoting genetic and epigenetic changes critical to CRC pathogenesis. Chronic inflammation driven by microbial imbalances activates pathways such as NF-κB and β-catenin, which support sustained cell proliferation, immune evasion, and metastasis (De Filippo et al., 2024; Masdor et al., 2022). The synergistic effects of high junk food consumption, gut dysbiosis, and exposure to dietary carcinogens create a feedback loop of inflammation, oxidative stress, and DNA damage. This intricate interaction underscores the vital roles of diet and the gut microbiome in CRC development, underscoring the importance of addressing dietary habits and gut health as pivotal components of CRC prevention strategies (Shah et al., 2023; Osborn et al., 2021).

Physical activity has been shown to significantly influence the gut microbiota, with potential implications for CRC prevention and progression. Regular exercise modifies gut microbial composition by increasing the abundance of beneficial bacteria, enhancing microbial diversity, and boosting the production of short-chain fatty acids (SCFAs), such as butyrate, which have anti-inflammatory and antitumorigenic properties. SCFAs enhance intestinal barrier integrity, regulate immune function, and inhibit the proliferation of pathogenic bacteria, such as *F. nucleatum* and *Escherichia coli* strains harboring the pks genomic island, which are implicated in CRC development (Boytar et al., 2022; Wegierska et al., 2022). Exercise-induced changes in the microbiota counteract CRC-associated dysbiosis, characterized by decreased SCFA-producing bacteria and an overrepresentation of pro-inflammatory and tumorigenic species. For instance, SCFAs produced by gut microbes inhibit the expression of virulent genes in pathogenic bacteria, reduce oxidative stress, and protect against DNA damage and inflammation, both of which drive CRC progression. Furthermore, physical activity is associated with increased gut motility and improved metabolic health, helping maintain a favorable gut environment (Sun et al., 2022). However, the relationship between exercise intensity and its effects on microbiota remains complex. While moderate exercise promotes microbial diversity and anti-inflammatory properties, excessive or high-intensity exercise can increase gut permeability and inflammation, potentially negating the benefits of physical activity on the microbiome (Himbert et al., 2022; Monda et al., 2017).

The composition of the gut microbiome varies markedly between left-sided (LCC) and right-sided (RCC) colon cancers, influencing pathogenesis, clinical features, and therapeutic responses. Right-sided colon cancer, which originates in the proximal colon, is often associated with more advanced disease stages, a higher prevalence in older adults, and features such as microsatellite instability and CpG island methylation. It is enriched in microbiota, such as *Bifidobacterium dentium*, which exhibits a less invasive profile. In contrast, left-sided colon cancer is more common, often presents with earlier-stage symptoms, and is associated with chromosomal instability and VEGF-related pathways. Its microbiome is more diverse and includes pro-inflammatory taxa like *F. nucleatum* and *Clostridium perfringens*, which contribute to DNA damage and histone modifications. These microbiota distinctions not only correlate with differing molecular characteristics but also with therapeutic strategies, such as cetuximab for LCC and bevacizumab for RCC. Understanding the role of microbiota in these distinct carcinogenic pathways is crucial for tailoring targeted interventions (Suga et al., 2022; DeDecker et al., 2021; Zhong et al., 2020; Lansom et al., 2024). Dysbiosis, characterized by an imbalance in the composition or function of the gut microbiota, has emerged as both a driver and a consequence of colorectal tumorigenesis (Shen et al., 2025). Pathogenic enrichment, depletion of beneficial taxa, microbial metabolite dysregulation, and mucosal barrier disruption, when combined, provide a pro-inflammatory and genotoxic milieu that promotes malignant transformation. Recent research has shown that dysbiosis contributes to chronic inflammation, DNA damage, epigenetic modifications, and immune evasion, highlighting the microbiota as an active participant in CRC pathogenesis rather than a passive bystander (Rubinstein et al., 2013; Clay et al., 2022; Garrett, 2015). While numerous reviews have examined individual microbial species or virulence factors implicated in CRC, the present research is mostly descriptive and fragmented, with little integration across genomic, immunologic, and metabolic aspects. This has led to an incomplete understanding of how various microbial activities interact with host signaling pathways to affect tumor initiation, development, and molecular heterogeneity. To address this gap, the current review proposes a novel tri-axial mechanistic framework that unifies the major microbiota-driven pathways contributing to CRC. We propose that colorectal carcinogenesis is determined by three interconnected axes: (1) microbial genotoxicity coupled to cGAS-STING-mediated innate inflammation, (2) microbial metabolite-driven reprogramming of host receptors such as AhR, FXR, and TGR5, and (3) crypt-anchored biofilm formation that sustains IL-6/STAT3 activation and immune evasion.

This review critically synthesizes evidence supporting three interconnected microbial-host axes, namely cGAS-STING activation, metabolite-mediated signaling, and biofilm-driven inflammation, and explores their implications for molecular classification and therapy. To structure this narrative review, we first developed a conceptual outline dividing the topic into the major biological domains linking the gut microbiome to colorectal carcinogenesis, including inflammation, microbial genotoxicity, metabolite-driven signaling, biofilm-associated mechanisms, and their translational implications. Based on this framework, relevant literature was identified through searches of PubMed, Scopus, and Web of Science using combinations of keywords such as “colorectal cancer,” “gut microbiome,” “dysbiosis,” “inflammation,” “microbial metabolites,” “biofilms,” “cGAS-STING,” “AhR,” “FXR,” and “TGR5.” Priority was given to recent studies, mechanistic investigations, and clinically relevant reports, while seminal earlier studies were also included where necessary to provide foundational context. The selected literature was then evaluated for its relevance to microbiota-associated mechanisms involved in CRC initiation, progression, and molecular heterogeneity, with particular emphasis on inflammatory, metabolic, genotoxic, and biofilm-related pathways.

## 2 CRC epidemiology

According to GLOBOCAN data, the prevalence of CRC is approximately 10.5 %, meaning there are around 2 million individuals living with CRC worldwide. In the United Arab Emirates, the prevalence of CRC is higher than the global average, at around 10.2 %. These statistics underscore the importance of addressing CRC within our local context. Globally, the incidence of CRC is approximately 9.6 %, with approximately 2 million new cases diagnosed each year. In the United Arab Emirates, the incidence of CRC is approximately 10 %, resulting in around 550 new cases of CRC diagnosed in the UAE. CRC contributes to a significant number of deaths. The mortality rate for CRC is approximately 9.3 %, which is equal to an estimated 900,000 individuals worldwide. In the United Arab Emirates, the mortality rate due to CRC is approximately 10.3 %, resulting in 236 deaths due to CRC in the UAE (Global Cancer Observatory, 2022).

## 3 CRC risk factors

CRC arises from a multifaceted interaction among genetic predispositions, environmental influences, and microbial factors. While age and genetic predispositions, such as Lynch syndrome and familial adenomatous polyposis (FAP), remain important, new research points to the gut microbiota as a crucial determinant of CRC risk. Dysbiosis, characterized by disruptions in gut microbial communities, is increasingly recognized as central to the pathogenesis of CRC, particularly through its association with chronic inflammatory conditions. For instance, inflammatory bowel diseases (IBD), such as ulcerative colitis and Crohn's disease, exhibit distinct alterations in the gut microbiota linked to persistent intestinal inflammation, which significantly elevates the risk of developing CRC. Similarly, celiac disease and irritable bowel syndrome (IBS) disrupt the gut microbiota, which may promote a pro-inflammatory environment and epithelial barrier failure, increasing susceptibility to CRC (Mori and Pasca, 2021; Kim and Lee, 2021).

## 4 Gut microbiome and dysbiosis

The human colon hosts one of the body's densest and most diverse microbial ecosystems, comprising bacteria, archaea, viruses, and fungi that collectively outnumber human cells. In a healthy state, this eubiotic microbiota is dominated by the phyla Firmicutes and Bacteroidetes, with smaller contributions from Actinobacteria, Proteobacteria, and Verrucomicrobia. These commensal communities support epithelial integrity, immune maturation, and metabolic homeostasis by producing essential metabolites, particularly short-chain fatty acids (SCFAs) such as butyrate and synthesizing vitamins including B and K. Beneficial taxa such as *Faecalibacterium, Roseburia, Lactobacillus*, and *Bifidobacterium* reinforce tight junctions, suppress inflammation, and protect against pathogens through competitive exclusion and antimicrobial peptide production (Ionescu et al., 2025; Shakhpazyan et al., 2024; Rinninella et al., 2019; DeGruttola et al., 2016; Flandroy et al., 2018). Dysbiosis, defined as a disruption in the composition and function of the gut microbiota, has emerged as a pivotal factor in CRC pathogenesis. CRC-associated dysbiosis is characterized by depletion of SCFA-producing commensals and enrichment of pathogenic species, including pks-Escherichia coli, enterotoxigenic Bacteroides fragilis (ETBF), and *Fusobacterium nucleatum* (Fusco et al., 2023; Artemev et al., 2022). These organisms use various virulent mechanisms that directly contribute to tumor formation and development. Genotoxic E. coli strains produce colibactin, a DNA-alkylating chemical that causes double-strand breaks and genomic instability (Artemev et al., 2022). ETBF secretes BFT, which cleaves E-cadherin and activates signaling pathways including β-catenin, NF-κB, and STAT3 (Lee et al., 2022). F. nucleatum expresses adhesins such as *FadA*, which binds to E-cadherin to activate Wnt/β-catenin, and *Fap2*, which interacts with the immune receptor TIGIT, allowing immune evasion (Schöpf et al., 2025). Together, these dysbiotic shifts impair epithelial barrier integrity, increase intestinal permeability, and activate innate immune sensors, including Toll-like receptors. This chronic activation of inflammatory pathways increases mucosal inflammation, biofilm formation, and immunological regulation, resulting in a tumor-promoting milieu favorable to CRC development. These microbial imbalances initiate multiple host responses that can be organized into three mechanistically distinct but overlapping axes.

## 5 Inflammation and its role in CRC

**Figure 1:**
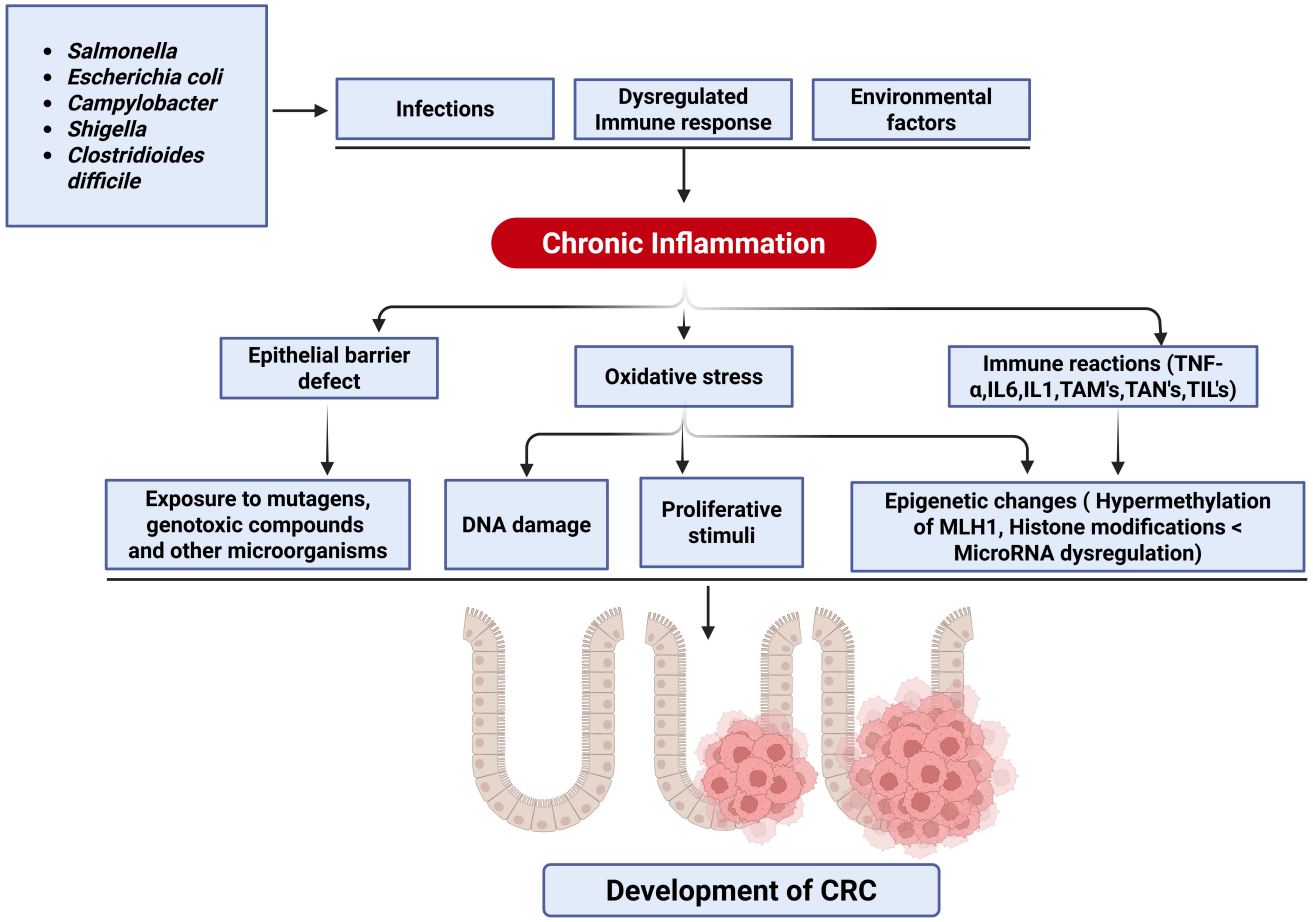
Mechanistic overview of chronic inflammation-driven colorectal cancer (CRC) development. This diagram outlines the molecular pathways through which chronic inflammation facilitates colorectal carcinogenesis. Chronic inflammation arises from persistent infections caused by pathogens such as *Salmonella, Escherichia coli, Shigella*, and *Clostridium difficile*, as well as from dysregulated immune responses and environmental factors. Inflammation-induced epithelial barrier dysfunction increases exposure to microbial toxins, genotoxic compounds, and mutagens, leading to direct DNA damage. Simultaneously, persistent oxidative stress is induced by chronic inflammatory mediators, including tumor necrosis factor-alpha (TNF-α), interleukin-6 (IL-6), interleukin-1 (IL-1), tumor-associated macrophages (TAMs), tumor-associated neutrophils (TANs), and tumor-infiltrating lymphocytes (TILs), promotes DNA mutations and abnormal cellular proliferation. Epigenetic modifications, such as hypermethylation of mismatch repair genes [e.g., MutL homolog 1 (MLH1)], histone alterations, and microRNA dysregulation, further exacerbate genomic instability and cancer progression. Collectively, these molecular events sustain a pro-carcinogenic environment, ultimately resulting in colorectal tumorigenesis.

Collectively, dysbiosis fosters a pro-tumorigenic microenvironment by disrupting mucosal homeostasis and promoting inflammation, genomic instability, and neoplastic transformation (Shen et al., 2025; Zou et al., 2018). Inflammation is a double-edged sword: necessary for tissue healing and infection defense, yet harmful if left untreated. Chronic, unresolved inflammation, referred to as “smoldering inflammation,” creates a tumor-promoting environment by releasing cytokines, chemokines, growth factors, and reactive oxygen/nitrogen species (ROS/RNS), leading to DNA damage and genomic instability (Cervantes-Villagrana et al., 2014). Chronic inflammation in the tumor microenvironment alters immune responses. Tumor-associated macrophages (TAMs) develop a pro-tumorigenic M2 phenotype, with increased VEGF and TGF-β promoting immune evasion and tumor vascularization. Disrupting the intestinal epithelial barrier through inflammation or microbial action allows environmental mutagens and microbial infiltration, exposing stem cells to genotoxic compounds (Shao et al., 2024). Dysbiosis in the colorectal mucosa initiates tumor development by persistently activating inflammatory pathways, including NF-κB and STAT3. The disruption of epithelial junctions increases permeability, allowing microbial products to penetrate the mucosa and activate innate immune sensors (Bunt et al., 2009). Figure 1 illustrates a mechanistic overview of chronic inflammation-driven CRC development.

Dysbiosis is a key initiator of CRC progression by triggering chronic inflammation through various virulence factors that can disrupt host signaling pathways, promote DNA damage, and alter immune responses. Host *TLR* gene mutations and polymorphisms like *TLR2, TLR4*, and *TLR5* can lead to impairment in microbial sensing and barrier function that will foster immune dysregulation and dysbiosis, this allows colonization by pathogens such as *E.coli, Bacteroides fragilis*, and *F. nucleatum*, which in turn drive chronic NF-κB and STAT3 activation, culminating in a sustained inflammatory environment conducive to colorectal tumorigenesis (Pandey et al., 2023). Several pathogenic bacteria have acquired virulent factors that exploit and sustain inflammatory reactions, thereby directly contributing to colorectal carcinogenesis. *E.coli* strains with the pks genomic island produce colibactin, a genotoxin that induces DNA double-strand breaks, activates DDR, and persistently triggers NF-κB, STAT3, and Wnt/β-catenin signaling, ultimately promoting proliferation, survival, and immune evasion (Rosendahl Huber et al., 2024; Tariq et al., 2022). *Enterotoxigenic Bacteroides fragilis* secretes BFT, which cleaves E-cadherin and releases β-catenin to promote oncogenic gene expression. It also activates NF-κB and STAT3. This results in ongoing *IL-6* and *IL-8* production, forming a pro-inflammatory microenvironment. Several microbial and viral infections have been linked to the development and progression of CRC via various molecular mechanisms. These organisms not only damage epithelial integrity but also activate oncogenic and inflammatory signaling pathways, thereby promoting cancer. The next section focuses on major microbial virulence factors and their associated pathways, emphasizing how bacterial and viral infections lead to chronic inflammation, genomic instability, immune evasion, and, ultimately, CRC formation. *F. nucleatum* contributes via several mechanisms: i) *FadA* activates Wnt signaling by binding to E-cadherin. ii) *Fap2* suppresses immune responses by interacting with TIGIT on T and NK cells. RadD activates PI3K-AKT-NF-κB-MMP9 pathways by engaging CD147. Annexin A1, which is increased by infection, promotes β-catenin signaling and epithelial transformation (Dadgar-Zankbar et al., 2023; Zepeda-Rivera et al., 2024). *Helicobacter pylori* can contribute to CRC progression predominantly through its virulence proteins *CagA* and *VacA*. (i) *CagA* enters epithelial cells via a type IV secretion system, phosphorylates and activates SHP-2, resulting in abnormal Ras-ERK and Wnt/β-catenin signaling. This promotes nuclear β-catenin accumulation and transcription of oncogenes such as *c-MYC* and *cyclin D1*. *CagA* promotes NF-κB and STAT3 pathways, which promote pro-inflammatory cytokine expression (e.g, *IL-8, TNF-*α), survival signaling (*Bcl-2, Bcl-xL*), and immune evasion. (ii) *VacA* causes mitochondrial malfunction, generates ROS, activates NF-κB, and promotes chronic inflammation and epithelial damage (Nguyen et al., 2024; Liu Y. et al., 2024). *Campylobacter jejuni* induces CRC via its genotoxin, the cytotoxin CDT.CdtB activates the ATM/ATR-mediated DNA damage response (DDR) and the IKK complex, leading to NF-κB activation. This causes chronic *IL-6* and *TNF-*α production, as well as G2/M cell cycle arrest, leading to genomic instability. Furthermore, *C. jejuni* alters the host's gut microbiota and activates the mTOR pathway, which promotes epithelial proliferation and tumor formation (He et al., 2019, 2024). Furthermore, viral infections such as norovirus and severe acute respiratory syndrome coronavirus 2 (SARS-CoV-2) have also been linked to alterations in the microbiota. Norovirus reduces microbial diversity and impairs gut barrier function, thereby triggering systemic inflammation via TLR activation. SARS-CoV-2 exacerbates dysbiosis by targeting ACE2-expressing enterocytes, reducing anti-inflammatory bacteria such as *Faecalibacterium prausnitzii*, enriching pathogenic species such as *Fusobacterium nucleatum*, and increasing cytokines like *IL-6*. This chronic inflammatory state elevates CRC risk, particularly through the gut–lung axis, which facilitates immune-mediated microbial dysregulation and tumor-promoting inflammation (Odun-Ayo and Reddy, 2022). Thus, chronic inflammation serves as a critical biological link between microbial dysbiosis and CRC. The following sections look at how unique microbial activities, such as genotoxicity (Axis I), metabolite-driven receptor reprogramming (Axis II), and crypt-anchored biofilms (Axis III), activate diverse inflammatory and oncogenic pathways which act together to induce colorectal tumorigenesis.

## 6 Key pathways linking microbiota and CRC

### 6.1 Microbiota-driven inflammatory signaling

**Figure 2:**
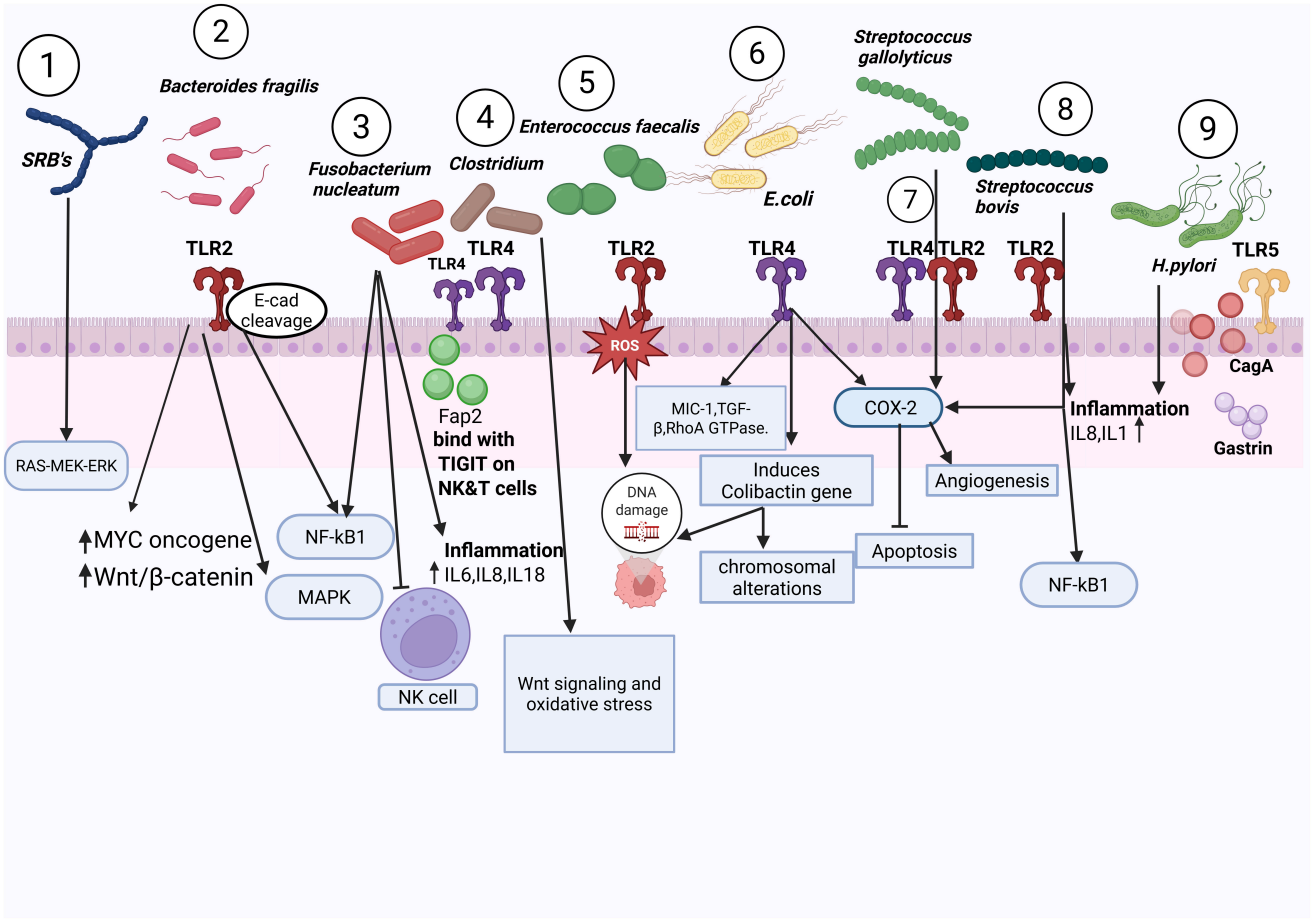
Microbial interactions and molecular signaling pathways implicated in CRC progression. This schematic highlight key microbial species and their molecular mechanisms that contribute to colorectal carcinogenesis. (1) Sulfate-reducing bacteria (SRBs) stimulate the RAS–MEK–ERK pathway, promoting MYC activation and enhancing Wnt/β-catenin signaling. (2) *Bacteroides fragilis* activates TLR2, cleaves E-cadherin, and induces MYC and NF-κB signaling. (3) *F. nucleatum* interacts via TLR4 and its Fap2 virulence factor, binding TIGIT on NK and T cells to suppress immunity and elevate IL-6, IL-8, and IL-18 production. (4) *Clostridium* species activate inflammatory pathways through TLR4. (5) *Enterococcus faecalis* generates ROS via TLR2, inducing DNA damage and promoting MIC-1, TGF-β, and RhoA signaling. (6) Genotoxic *E. coli* expressing colibactin activates TLR4, causes DNA damage, and promotes chromosomal instability. (7–8) *Streptococcus gallolyticus* and *S. bovis* trigger COX-2-driven angiogenesis, suppress apoptosis, and amplify inflammation via TLR2/4. (9) *Helicobacter pylori* engage TLR5, secretes CagA, and induces gastrin, promoting chronic inflammation via elevated IL-8 and IL-1. Collectively, these pathways illustrate how microbiota-driven signaling fosters immune evasion, DNA damage, and tumor progression in CRC.

Microbial dysbiosis promotes CRC by coordinating the activation of inflammatory and oncogenic signaling pathways that remodel both epithelial and immune cell behavior. Numerous pathogenic taxa, including *Bacteroides fragilis, Fusobacterium nucleatum, Enterococcus faecalis, Clostridium, Streptococcus gallolyticus*, and *H. pylori*, engage host pattern-recognition receptors, particularly Toll-like receptors, triggering NF-κB and STAT3 activation and driving sustained cytokine production, including IL-6, IL-8, and IL-18. These inflammatory circuits establish a chronic mucosal state that favors epithelial proliferation and survival. In parallel, specific microbial factors directly modulate oncogenic pathways: *B. fragilis* disrupts E-cadherin and activates Wnt/β-catenin; *F. nucleatum* promotes NF-κB signaling and suppresses NK-cell cytotoxicity via Fap2–TIGIT interactions; and *E. faecalis* generates reactive oxygen species that induce DNA damage and chromosomal instability, further enhanced by colibactin-producing *E. coli*. Additional bacterial signals stimulate COX-2, thereby fostering angiogenesis and resistance to apoptosis. Collectively, Figure 2 illustrates how dysbiosis-driven activation of innate immune receptors, oncogenic signaling pathways, and DNA-damaging processes converge to promote epithelial transformation, immune evasion, and genomic instability, thereby driving colorectal carcinogenesis (Santaolalla et al., 2013; Villar-Ortega et al., 2021; Iftekhar et al., 2021; Novellasdemunt et al., 2015; Liu C. et al., 2024; Bennedsen et al., 2022).

### 6.2 Interaction between microbiome and immune cells

**Figure 3:**
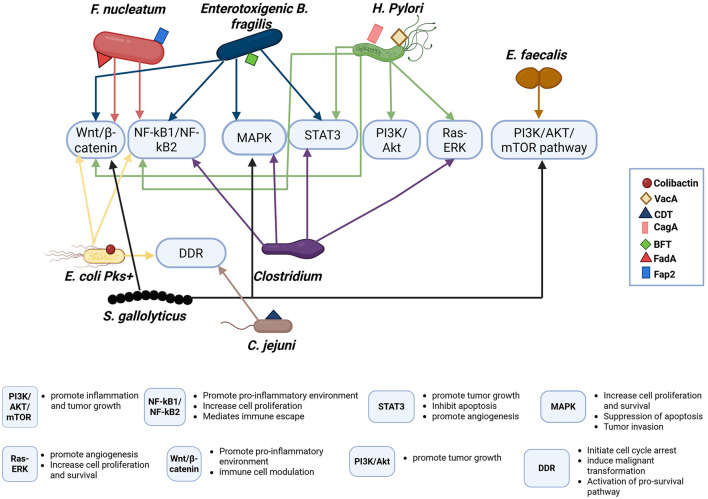
Microbial virulence factors and the host signaling pathways in colorectal carcinogenesis. The figure below illustrates interactions between colorectal cancer (CRC) and bacteria, as well as the host signaling pathways they stimulate. *Fusobacterium nucleatum* (FadA, Fap2) activates Wnt/β-catenin and NF-κB signaling, leading to inflammation, immunological evasion, and epithelial proliferation. *Enterotoxigenic Bacteroides fragilis* (BFT) activates the MAPK and STAT3 pathways, thereby promoting IL-6-induced proliferation and angiogenesis. *Helicobacter pylori* (CagA, VacA) activate the Ras-ERK and PI3K/AKT/mTOR signaling pathways, promoting tumor growth and cell survival. *Escherichia coli* (pks^+^) produces colibactin, which triggers the DNA damage response and genomic instability. *Campylobacter jejuni* (CDT) also induces DDR, leading to cell cycle arrest and malignant transformation. *Streptococcus gallolyticus* activates NF-κB and MAPK, increasing proliferation and inflammation, while *Clostridium* species increase oxidative stress and DNA damage. *Enterococcus faecalis* activates PI3K/AKT/mTOR signaling via ROS production and TLR2 activation, which reinforces inflammatory and pro-survival responses.

Under normal conditions, gut microbiota interacts with host immunity through microbial-associated molecular patterns, which are sensed by Toll-like receptors and other pattern recognition receptors. In dysbiosis, however, increased exposure to bacterial ligands drives excessive activation of pathways such as NF-κB, STAT3, PI3K/Akt, Ras-ERK, and Wnt/β-catenin-many of which are shown in Figure 2 to be stimulated by virulence factors from *F. nucleatum, Enterotoxigenic Bacteroides fragilis, E. coli pks*^+^*, H. pylori, E.s faecalis, C.jejuni*, and *S.gallolyticus*. This signaling imbalance enhances cytokine production, recruits tumor-associated macrophages, and suppresses cytotoxic immune responses, progressively weakening mucosal immune surveillance. Dysbiosis simultaneously promotes the formation of mucosal biofilms that adhere tightly to the epithelial surface, disrupt barrier integrity, and create continuous microbial-epithelial contact that further amplifies inflammatory and oncogenic signaling. Figure 3 shows how this combined activation of inflammatory pathways, epithelial damage, immune evasion, and microbial persistence establishes a pro-tumorigenic immune microenvironment that accelerates colorectal cancer development and progression (He et al., 2024; Odun-Ayo and Reddy, 2022; Santaolalla et al., 2013; Villar-Ortega et al., 2021; Iftekhar et al., 2021; Novellasdemunt et al., 2015).

### 6.3 Dysbiosis, leaky gut, and inflammation

**Figure 4:**
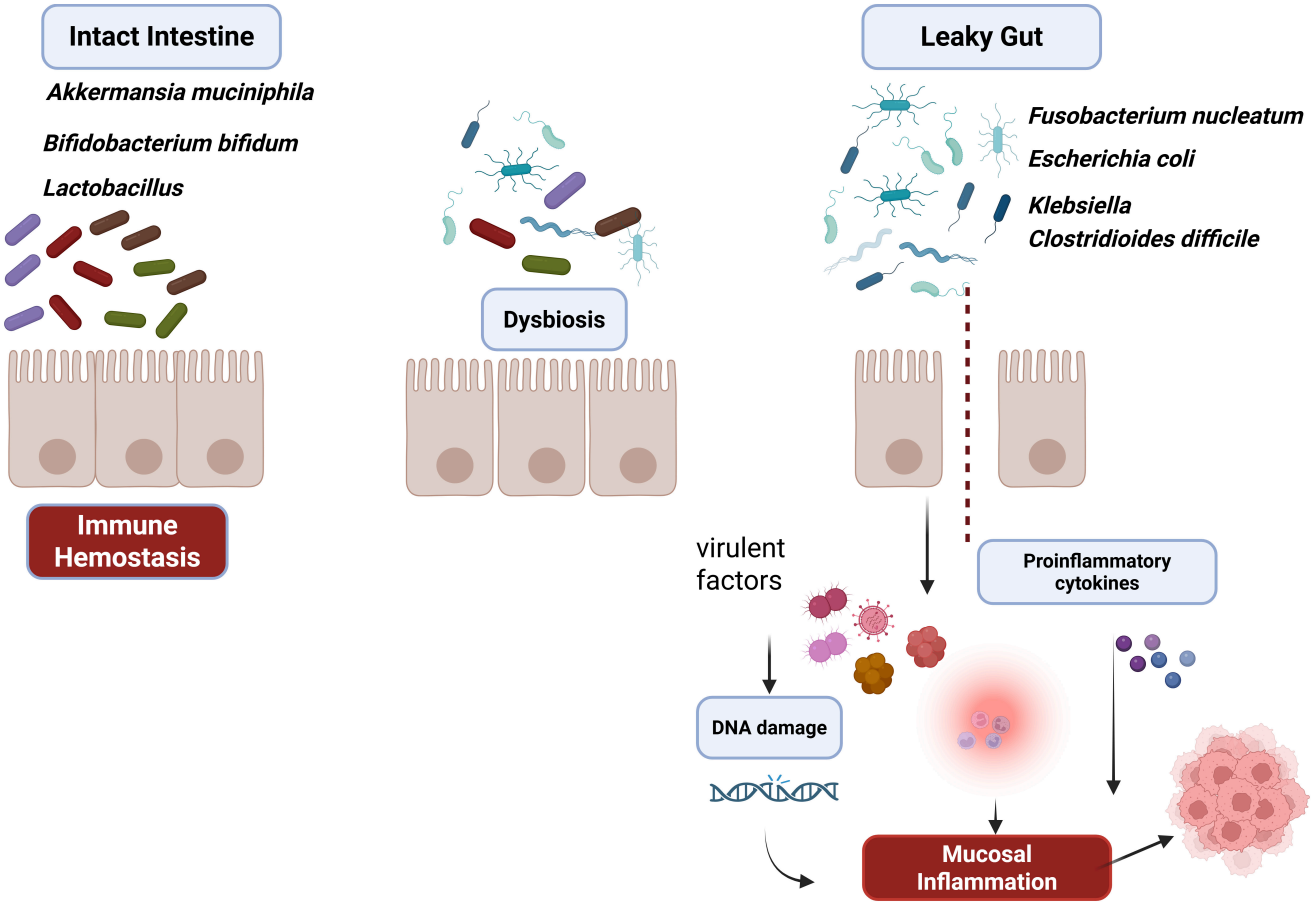
Leaky gut and colorectal cancer molecular and microbial pathogenesis: This diagram illustrates the progression from an intact intestinal epithelium to a leaky gut state that contributes to CRC. Under normal conditions, beneficial bacteria such as *Bifidobacterium bifidum* and *Akkermansia muciniphila* maintain tight junction integrity and immune balance. Dysbiosis, characterized by the depletion of protective microbes and the expansion of pathogenic species such as *Escherichia coli* and *F. nucleatum*, leads to the release of virulent factors that disrupt epithelial barriers and increase permeability. This facilitates the translocation of microbial components, triggering immune activation, pro-inflammatory cytokine release, and the production of reactive oxygen species (ROS). These events induce DNA damage, mucosal inflammation, and activate oncogenic signaling, ultimately driving epithelial–mesenchymal transition (EMT) and tumor progression.

Increased intestinal permeability, sometimes known as “leaky gut,” exacerbates these immunological abnormalities, which are a key consequence of dysbiosis, characterized by the loss of beneficial bacteria and the expansion of pathogenic species. This “leaky gut” allows microbial compounds to translocate and trigger epithelial and immunological pathways, resulting in a feed-forward loop of inflammation, oxidative stress, and epithelial injury. Persistent barrier dysfunction leads to genomic instability, epigenetic alterations, and a milieu favorable to CRC growth. When the epithelial barrier is compromised, microbial products, including LPS, peptidoglycans, and virulence factors, translocate across the mucosa and engage epithelial and immune receptors, triggering NF-κB and STAT3 activation, cytokine release, and persistent immune cell recruitment. This feed-forward loop of inflammation, oxidative stress, and epithelial injury further weakens barrier function, allowing even greater microbial penetration. Over time, chronic exposure to these inflammatory and genotoxic stimuli leads to DNA damage, genomic instability, and epigenetic alterations that silence tumor-suppressor genes, collectively creating a microenvironment favorable to CRC development and progression, as shown in Figure 4 (Liu C. et al., 2024; Bennedsen et al., 2022; Nishida and Andoh, 2025).

## 7 Microbiota-induced pathogenesis of CRC

Following prolonged inflammatory reactions caused by microbial dysbiosis, CRC develops as a final pathological outcome influenced by both host genetic susceptibility and microbial-driven immune dysregulation. Dysbiosis, marked by the overgrowth of pathobionts such as *F. nucleatum, E. coli (pks*^+^*)*, and *Enterotoxigenic Bacteroides fragilis*, leads to intestinal inflammation by disrupting epithelial tight junctions, activating TLR pathways, and increasing cytokine production. The inflammatory microenvironment is sustained by NF-κB, STAT3, and Wnt/β-catenin signaling pathways, which promote epithelial cell proliferation, immune evasion, and genomic instability (Nishida and Andoh, 2025; Giambra et al., 2023). These microbe-induced changes create a pro-tumorigenic environment, setting the mucosa for neoplastic transformation. In its early stages, chronic inflammation produces ROS/RNS, causing DNA damage and epigenetic alterations. These include hypermethylation of tumor suppressor genes such as MLH1 and p16INK4a, and activation of oncogenes such as c-MYC and KRAS, processes similar to classical CRC pathways, including chromosomal instability (CIN), microsatellite instability (MSI), and the CpG island methylator phenotype (CIMP) (Clay et al., 2022; Lee et al., 2022; Rubinstein et al., 2013).

**Figure 5:**
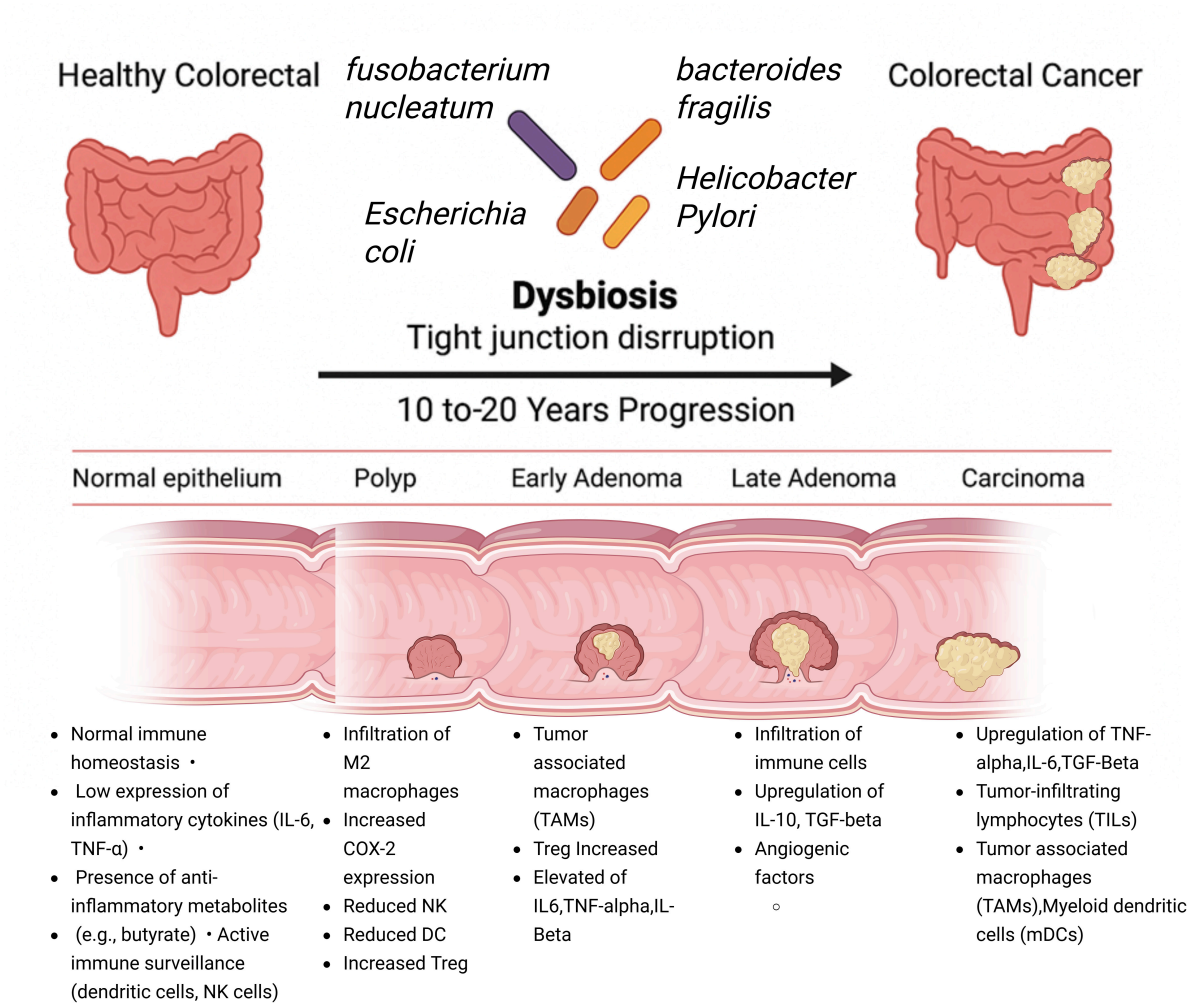
Microbiota-induced inflammation drives colorectal cancer progression via molecular and immune dysregulation. This diagram illustrates the gradual progression from normal epithelium to CRC over a 10- to 20-year period. Dysbiosis, characterized by the expansion of pathobionts such as *Fusobacterium nucleatum, Escherichia coli (pks*^+^*)*, enterotoxigenic *Bacteroides fragilis* (ETBF), and *Helicobacter pylori*, leads to the disruption of epithelial tight junctions and activation of pro-inflammatory signaling pathways, including nuclear factor-kappa B (NF-κB), signal transducer and activator of transcription 3 (STAT3), and Wnt/β-catenin. This chronic inflammatory environment induces DNA damage and epigenetic alterations, such as hypermethylation of the MutL homolog 1 (MLH1) gene, promoting classical CRC pathways including chromosomal instability (CIN), microsatellite instability (MSI), and the CpG island methylator phenotype (CIMP). Key mutations in adenomatous polyposis coli (APC), Kirsten rat sarcoma virus (KRAS), and tumor protein p53 (TP53), along with immune evasion mechanisms involving regulatory T cells (Tregs) and tumor-associated macrophages (TAMs), facilitate the transition from polyp to adenoma to carcinoma, ultimately linking host genetic susceptibility to microbial-induced inflammation in CRC pathogenesis.

CRC development typically follows a well-defined adenoma-carcinoma cycle over 10–15 years, with four critical stages: initiation, promotion, progression, and metastasis. Host genetics influences this transition, and microbial virulence factors also play an important role. Microbial agents such as colibactin from *pks*+ E. coli, *BFT* from Bacteroides fragilis, and *FadA* from F. nucleatum act as biological initiators by causing DNA double-strand breaks, disrupting E-cadherin-dependent adhesion, and persistently activating pro-inflammatory and oncogenic signaling pathways. As these microbial effects persist, they combine with the underlying genetic and epigenetic changes that cause cancer. Mutations in the APC gene, seen in 80% of CRCs, activate the Wnt pathway and accumulate nuclear β-catenin, leading to uncontrolled cell proliferation. *KRAS* mutations, found in 50–60% CRC cases, promote proliferative signaling via the RAF-MEK-ERK and PI3K-AKT-mTOR cascades. In later stages, *TP53* mutations disrupt apoptotic systems, allowing genetically unstable cells to survive and accumulate. In addition, defects in mismatch repair genes such as *MLH1, MSH2, and MSH6*, frequently caused by promoter hypermethylation, contribute to the microsatellite instability (MSI) phenotype and are significantly associated with the CpG island methylator phenotype (CIMP), particularly in serrated neoplasia. These molecular abnormalities work together to create a favorable environment for colorectal tumor initiation and progression in the presence of chronic inflammatory microbial dysbiosis (Novellasdemunt et al., 2015; Coretti et al., 2024; Dawe and Di Meglio, 2025; Santana et al., 2024; Yu et al., 2025). CRC is now understood as a highly heterogeneous disease. Chromosomal instability (CIN)-driven tumors (80–85%) exhibit aneuploidy, chromosomal missegregation, and loss of heterozygosity, whereas MSI-high tumors (15%) exhibit hypermutation and are frequently associated with BRAF mutations and serrated neoplasia pathways (Chen et al., 2018; Song et al., 2018). In this context, the gut microbiota is a dynamic regulator of the tumor microenvironment, influencing host immune responses, genetic stability, and tumor subtype evolution. As a result, the gut microbiota plays a critical role in the multistep etiology of CRC and is also changeable. Figure 5 illustrates how microbiota-induced inflammation drives CRC progression through a network of molecular and immune dysregulation. Together, these processes illustrate the broad ways in which the microbiome influences host signaling. These diverse microbial effects converge mechanistically into three dominant pathogenic axes, detailed below.

### 7.1 Axis I: microbial genotoxicity and cGAS-STING-mediated inflammation

**Figure 6:**
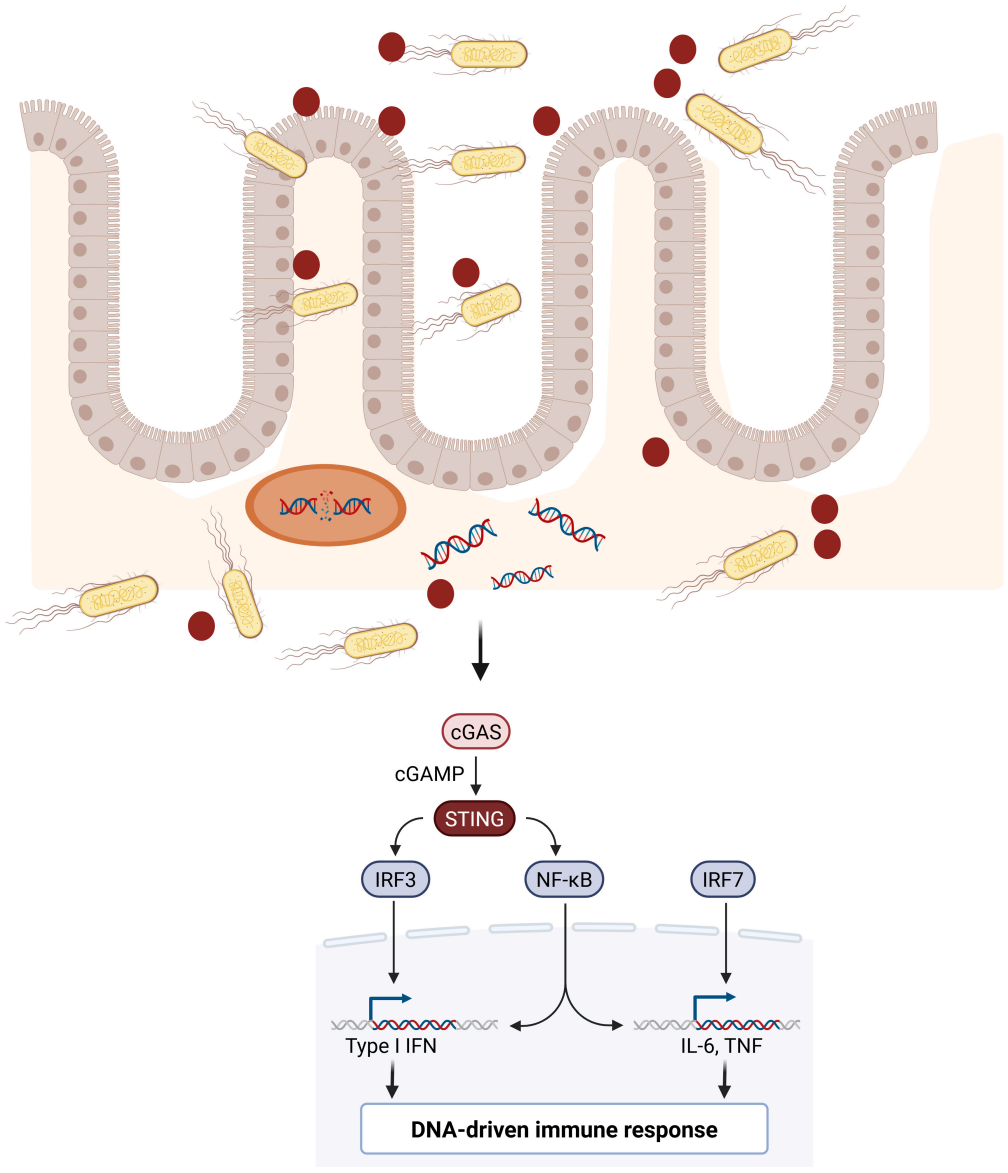
Microbial genotoxicity activates cGAS–STING signaling in colorectal cancer. This diagram illustrates that Colibactin-producing *Escherichia coli* (pks^+^ *E. coli*) induces DNA interstrand crosslinks and double-strand breaks in colonic epithelial cells, leading to replication stress, genomic instability, and the formation of micronuclei. Rupture of micronuclei releases host DNA into the cytosol, where it is sensed by cGAS, triggering production of 2′3′-cGAMP and activation of STING at the endoplasmic reticulum. STING signaling activates TBK1 and NF-κB/IRF3 pathways, inducing type I interferons and pro-inflammatory cytokines. While transient cGAS–STING activation supports antitumor immunity, chronic activation sustains inflammation, epithelial survival, and immune evasion, thereby promoting colorectal cancer progression.

Axis I describes how microbial genotoxins initiate DNA damage and innate immune sensing through the cGAS-STING pathway. Colibactin-producing Escherichia coli (pks^+^ E. coli) are highly investigated genotoxic bacteria linked to CRC. The pks genomic island encodes a 54-kb hybrid nonribosomal peptide-polyketide synthetase (NRPS-PKS) machinery that produces colibactin, a small-molecule genotoxin that alkylates host DNA. This biosynthetic cluster generates intermediates with a reactive cyclopropane ring that covalently binds adenine residues on opposing DNA strands. Infected epithelial cells with pks^+^ E. coli exhibit DNA double-strand breaks (DSBs) due to crosslinks that halt replication forks, characterized by strong γ-H2AX accumulation and ATM/ATR activation (Xi et al., 2021). Adenine-rich trinucleotide mutations produced by colibactin have been found in human CRCs, suggesting a relationship between pks^+^ E. coli exposure and endogenous cancer mutations (Xi et al., 2021). *In-vivo* studies confirm that colibactin is required for tumor promotion. Mice colonized with pks^+^ strains show greater epithelial injury, increased DSBs, and enhanced tumor multiplicity, whereas isogenic pks^+^ mutants fail to induce these phenotypes (Gutierrez-Angulo et al., 2023). Mechanistic studies further reveal that colibactin-induced DSBs trigger a persistent DNA damage response (DDR), including ATM/ATR signaling, G2/M arrest, chromosomal segregation abnormalities, and long-term genomic instability, all of which are critical steps in early CRC formation (Rubinstein et al., 2013). Furthermore, pks island expression has been linked to CRC susceptibility in human populations, demonstrating its importance beyond experimental paradigms (Antonangeli et al., 2020). Colibactin's severe DNA damage causes fragmented host DNA to accumulate in the cytosol, where it activates the cGAS-STING innate immune pathway. The cGAS-STING pathway is a key innate immune surveillance system for detecting abnormal DNA in the cytosol. Under normal circumstances, host DNA is restricted to the nucleus and mitochondria; thus, the presence of double-stranded DNA (dsDNA) in the cytoplasm serves as a warning signal. The enzyme cGAS (cyclic GMP-AMP synthase) binds to cytosolic dsDNA, whether from pathogens or damaged host nuclei, and synthesizes the second messenger 2′3′-cGAMP, which activates STING (stimulator of interferon genes) in the endoplasmic reticulum. Beyond directly producing mutational signatures, colibactin-mediated double-strand breaks, and other types of genomic instability, produce mislocalized DNA fragments and micronuclei that can be detected by the cytosolic DNA receptor cGAS. When cGAS binds to microbial or damaged self-dsDNA, it synthesizes the second messenger 2′3′-cGAMP. This activates the adaptor STING on the endoplasmic reticulum, leading to the activation of TBK1 and IKK, phosphorylation of IRF3 and NF-κB, and induction of type I interferons and inflammatory cytokines that bridge innate and adaptive immunity, as illustrated in Figure 6 (Betzler et al., 2020; Galaski et al., 2021).

In the gut, the cGAS-STING pathway can be activated through a variety of biologically distinct pathways that link microbial dysbiosis to early tumor immunity. During dysbiosis, breakdown of epithelial tight junctions and mucus layers promotes microbial translocation, allowing bacterial DNA, cyclic dinucleotides, and other pathogen-associated signals to reach the cytosol of epithelial and immune cells. Genotoxic exposure to pks^+^ E. coli causes DNA double-strand breaks and the formation of micronuclei. Rupture of these micronuclei releases substantial amounts of host-derived DNA into the cytoplasm, which is readily detected by cGAS. During therapy-induced genotoxic stress, additional sources of cytosolic DNA emerge, including radiation and certain chemotherapeutic drugs, which produce fragmented nuclear and mitochondrial DNA, further activating the cGAS-STING machinery. When cGAS binds to these aberrant DNA fragments, it produces 2′3′-cGAMP, which activates STING and results in a strong innate immune response. STING signaling promotes dendritic cell maturation, antigen cross-presentation, and recruitment of cytotoxic CD8^+^ T lymphocytes that eliminate injured or pre-malignant epithelial cells, initially suppressing tumor growth. Thus, cGAS-STING acts as a critical link between microbial genotoxic stress, innate immune sensing, and early antitumor immunity, laying the groundwork for its context-dependent role in CRC progression (Schöpf et al., 2025; Duizer et al., 2025). However, new research indicates that persistent or dysregulated cGAS-STING signaling can also sustain protumorigenic inflammation and immune exhaustion, highlighting its context-dependent function in colorectal carcinogenesis, which will be discussed further in the next subsections (Gur et al., 2019; Maiuri et al., 2019). When cGAS identifies cytosolic DNA and activates STING, the complex sets off a downstream signaling cascade that links genotoxic stress to inflammatory and carcinogenic programs. Activated STING recruits and activates TANK-binding kinase 1 (TBK1), which phosphorylates IRF3, causing dimerization and nuclear translocation to induce type I interferons and interferon-stimulated genes that shape the early innate response (Betzler et al., 2020; Allen et al., 2019). STING activation stimulates the IKK complex, leading to IκB degradation and nuclear translocation of NF-κB. This upregulates pro-inflammatory cytokines such as IL-6, IL-1β, and TNF-α, as well as chemokines (Betzler et al., 2020; Li Z. et al., 2025). Chronic activation of the TBK1-IRF3-NF-κB axis in the gastrointestinal tract, especially the colon, has been linked to persistent mucosal inflammation and tissue remodeling in digestive diseases such as CRC (Li W. J. et al., 2025). Sustained NF-κB activation promotes epithelial proliferation, resistance to apoptosis, and a cytokine-rich microenvironment that promotes survival and clonal expansion of cells injured by microbial genotoxins such as colibactin (Lai et al., 2019). Meanwhile, sustained TBK1-IRF3 activity contributes to metabolic stress, immune-cell recruitment, and modification of the tumor microenvironment, progressively transforming cGAS-STING from an immediately protective, tumor-suppressive system to a chronic pro-tumor inflammatory driver. The cGAS–STING axis has context-dependent dual functions in CRC. Early activation of cGAS-STING suppresses tumors by triggering type I interferons, immunogenic cell death, and CD8^+^ T-cell recruitment. This maintains epithelial genome integrity and promotes effective antitumor immunity. Several studies have shown that transient STING activation limits the proliferation of cells containing microbial or damage-derived DNA and improves immune surveillance during the early stages of carcinogenesis (Thomas, 2012; Yeh et al., 2016). However, when cGAS-STING signaling becomes chronic, as is frequently the case with persistent microbial genotoxins, barrier leakage, or continuous DNA damage, the system shifts toward tumor-promoting inflammation. Sustained STING signaling activates NF-κB and induces the secretion of IL-6, TNF-α, and chemokines, thereby modifying the tumor microenvironment and promoting epithelial proliferation, survival, and immune evasion. Chronic activation also causes metabolic stress, epithelial senescence-associated secretory phenotypes (SASP), and the recruitment of myeloid suppressor cells, all of which contribute to CRC progression (Thomas, 2012; Lai et al., 2019). Thus, cGAS-STING acts as a double-edged sword. It is protective when activated temporarily, but it also drives chronic inflammation and tumor growth when persistently stimulated.

### 7.2 Axis II: microbial metabolites and host receptor reprogramming

Axis II explains how microbial metabolites reprogram epithelial and immune receptor signaling, influencing inflammation, metabolism, and epigenetic control. Microbial metabolites are a major link between dysbiosis and colorectal carcinogenesis. In healthy individuals, commensal bacteria ferment dietary fiber to produce short-chain fatty acids (SCFAs), particularly butyrate, which enhance epithelial energy metabolism, strengthen tight junctions, and reduce NF-κB-driven inflammation. CRC-associated dysbiosis is typically characterized by a decrease in SCFA-producing genera, resulting in reduced butyrate availability and loss of these protective benefits (Yang and Wang, 2025; Tomasi et al., 2006). Lactobacilli and other commensals produce tryptophan-derived indole compounds that activate AhR and stimulate IL-22 release while maintaining mucosal integrity. Dysbiosis affects indole synthesis and shifts AhR signaling toward pro-inflammatory or proliferative pathways, depending on ligand type and concentration (Lu et al., 2025; Zhang et al., 2024). Similarly, the proliferation of Clostridium species boosts the conversion of primary bile acids into secondary bile acids, including deoxycholic acid (DCA) and lithocholic acid (LCA). These compounds are genotoxic and activate FXR and TGR5, which promote oxidative stress, epithelial proliferation, and chronic inflammation (Kong et al., 2025; D'Ignazio et al., 2017). The aryl hydrocarbon receptor (AhR) is a ligand-activated transcription factor that integrates microbial, nutritional, and host-derived signals to control epithelial homeostasis and mucosal immunity. AhR has a context-dependent, dual role in CRC, functioning as both a barrier-protective factor and a driver of inflammation and tumor promotion, depending on ligand availability, microbial composition, and activation duration (Goldiş et al., 2025; Xu et al., 2022). AhR signaling has dual, context-dependent impacts on mucosal homeostasis and colorectal tumorigenesis. Under physiological conditions, commensal bacteria convert tryptophan into indole derivatives, such as indole-3-propionic acid and indole-3-aldehyde. These derivatives activate AhR, enhancing IL-22 production, promoting epithelial regeneration, strengthening tight junctions, and suppressing excessive NF-κB-driven inflammation. This helps maintain barrier integrity and prevent dysbiosis-associated injury (Coretti et al., 2024; Dawe and Di Meglio, 2025). Dysbiosis shifts the ligand pool toward high-affinity or inflammatory AhR agonists, such as kynurenine and dysregulated microbial indoles. This leads to chronic activation of STAT3 and NF-κB, increased epithelial proliferation, altered differentiation, and amplification of Wnt/β-catenin signaling. This chronic signaling alters cytokine networks, impairs immune surveillance, and promotes a tumor-friendly microenvironment. Thus, AhR acts as a ligand-specific molecular switch, protective when triggered by commensal metabolites but pro-tumorigenic when activated by dysbiosis-associated or inflammation-induced ligands (Santana et al., 2024; Yu et al., 2025).

The bile acid receptors FXR and TGR5 form a crucial protective axis that links microbial metabolism to CRC risk and understanding how their work clarifies their paradoxical behavior. FXR and TGR5 act as essential metabolic and immunological sensors, coordinating both local gut homeostasis and overall systemic balance via the gut-liver and gut-liver-brain axes. Within the intestine, activation of these receptors increases epithelial barrier integrity by regulating tight junction proteins, antimicrobial peptides, and mucus production, thereby reducing bacterial translocation and limiting exposure to inflammatory stimuli such as LPS (Lee et al., 2012). FXR and TGR5 inhibit NF-κB signaling in epithelial cells, macrophages, and dendritic cells, reducing local inflammation and promoting a healthy immune response to commensal microbiota. Bile acids circulating through the enterohepatic axis serve as signaling molecules for FXR and TGR5 in distant organs (Lee et al., 2012; Li W. J. et al., 2025). FXR activation in the ileum triggers FGF19 release, which travels to the liver and inhibits bile acid production via FGFR4, thereby maintaining metabolic stability. FXR also contributes to systemic homeostasis by decreasing triglycerides and cholesterol while increasing hepatic and adipose insulin sensitivity. TGR5 adds to these effects by activating GLP-1 release from intestinal L-cells, improving insulin secretion and glucose regulation, and increasing energy expenditure and adipose “browning,” which protects against obesity and metabolic dysfunction. FXR and TGR5 link gut microbial signals with endocrine and immunological networks via these interrelated pathways, ensuring both local intestinal integrity and overall metabolic and inflammatory homeostasis (Zhang et al., 2005; Allali et al., 2017; Coker et al., 2022). The transition from gut homeostasis to CRC is marked by a breakdown of the protective roles of *FXR* and *TGR5*, shifting these receptors from guardians of intestinal integrity to contributors to tumorigenesis through genetic, nutritional, and microbial disturbances (Aardema et al., 2021). *FXR*, a potent tumor suppressor, is significantly downregulated in human CRC, and low expression correlates with a poor prognosis. This loss is caused by mutations often identified in CRC, such as *APC* mutations, as well as by epigenetic silence that inhibits *FXR* expression. As FXR signaling decreases, its protective functions deteriorate. This leads to increased NF-κB-mediated inflammation, hyperactivity of the Wnt/β-catenin pathway (found in over 90% of CRCs), and weakening of the epithelial barrier, allowing bacterial products and inflammatory mediators to enter the mucosa and create a carcinogenic environment (John Kenneth et al., 2023; Romero-Garmendia and Garcia-Etxebarria, 2022; Louis et al., 2014). The gut microbiome promotes CRC progression by altering bile acid metabolism, thereby chronically activating the TGR5 receptor. Dysbiosis, particularly under high-fat diets, favors Bacteroides and Clostridium species with high bile salt hydrolase activity, thereby enhancing the conversion of primary bile acids into the potent TGR5 agonists deoxycholic acid (DCA) and lithocholic acid (LCA). Persistent activation of TGR5 by these secondary bile acids has pro-tumorigenic effects: in the colon, DCA and LCA stimulate the MAPK/ERK and PI3K/AKT pathways, enhancing cancer cell survival, proliferation, and resistance to apoptosis; in the liver, LCA activates TGR5 on cancer-associated fibroblasts, inducing chemokine secretion that recruits myeloid-derived suppressor cells and facilitates immune evasion during metastasis. Excess secondary bile acids also cause inflammation and decrease epithelial barrier defenses, promoting tumor growth (Rakoff-Nahoum and Medzhitov, 2007; Donohoe et al., 2012; Gur et al., 2015; Roy et al., 2025). The interaction of microbial metabolites and host epigenetic regulation in CRC occurs at a highly specific molecular level, primarily through modulation of key epigenetic enzymes and the availability of critical metabolic cofactors, establishing a direct biochemical link between dysbiosis and tumor-associated epigenetic remodeling. Microbial metabolites significantly influence epigenetic mechanisms in CRC by directly altering histone acetylation and DNA methylation. Commensals such as *Faecalibacterium prausnitzii* and *Roseburia intestinalis* produce the short-chain fatty acid butyrate, which enters colonocytes via MCT1 transporters and functions as a natural competitive inhibitor of Class I and II histone deacetylases (HDACs), especially *HDAC1*. Butyrate increases histone acetylation marks, such as *H3K9ac*, at tumor suppressor gene promoters by inhibiting HDAC activity. This improves chromatin accessibility and promotes normal epithelial differentiation and death. The Warburg metabolic shift in CRC cells reduces butyrate oxidation, leading to nuclear accumulation and more potent HDAC inhibition. This effect causes cancer cells to undergo apoptosis, but this is lost when dysbiosis lowers total butyrate production (Yu et al., 2024; Shang and Liu, 2018; Zhang et al., 2018; Chang et al., 2014). Through the one-carbon (SAM/folate) metabolic axis, the microbiota also affects DNA methylation. Bacterial contributions to folate availability support the conversion of homocysteine to methionine and the production of S-adenosylmethionine (SAM), the universal methyl donor for DNMTs. The SAM/SAH ratio is disrupted by dysbiosis or folate insufficiency, altering DNMT activity and contributing to the aberrant DNA methylation patterns typical of CRC. Furthermore, elevated levels of secondary bile acids, such as deoxycholic acid (DCA), induce oxidative stress and chronic inflammation, leading to lesions such as 8-oxo-dG in CpG-rich regions. DNMTs are then recruited to these damaged sites, leading to hypermethylation and silencing of important tumor suppressors, such as MLH1, thereby strengthening the CpG island methylator phenotype (CIMP) (Zhang et al., 2021; Kim, 2005). Together, these mechanisms reveal how microbial metabolites reshape the colon's epigenetic landscape, shifting it from a protective state to one that supports genomic instability and tumor progression. Crucially, the inconsistent results in the literature are explained by the highly context-dependent effects of FXR, TGR5, and microbial metabolites in CRC. When ligand concentrations, microbial composition, or metabolic states change, signals that are protective under normal conditions, such as butyrate-induced HDAC inhibition, TGR5-driven metabolic control, or FXR-mediated anti-inflammatory activity, can turn carcinogenic. These pathways can be inverted, from preserving barrier integrity and immunological balance to promoting inflammation, proliferation, and immune evasion due to prolonged exposure to toxic bile acids, changes in AhR ligand profiles, decreased butyrate synthesis, or loss of receptor expression. Therefore, the metabolic environment, microbial ecology, and disease stage determine whether these pathways prevent or promote carcinogenesis.

### 7.3 Axis III: crypt-anchored biofilms, IL-6/STAT3 signaling, and immune evasion

Axis III outlines how mucosal biofilms sustain IL-6/STAT3 signaling, disrupt epithelial integrity, and drive immune evasion. Biofilms frequently develop in the proximal (right-sided) colon and are strongly associated with CRC. These structured bacterial colonies adhere to the mucosal surface, cause chronic inflammation that promotes early carcinogenesis, damage the protective mucus layer, and enhance epithelial permeability. Clinical diagnosis primarily depends on biopsy-based techniques because these delicate structures can be removed by normal bowel preparation. Biofilms and their interactions with the epithelium can be visualized using high-definition endoscopy, fluorescence in situ hybridization (FISH), and sophisticated imaging methods, including confocal and electron microscopy. Biofilm-associated microorganisms are further identified using (Wang et al., 2021) molecular techniques such as PCR. Reliable non-invasive detection remains scarce despite the exploration of new strategies, including stool biomarkers and focused imaging, underscoring the need for better diagnostic techniques (Songtanin et al., 2022; Jandl et al., 2024; Anastasiadis et al., 2014). The mechanistic link between proximal colon biofilms and CRC is largely driven by persistent activation of the IL-6/STAT3 signaling cascade, which biofilms initiate by precise molecular disruptions of epithelial and immune pathways. In biofilm-rich mucosa, the metalloprotease BFT, secreted by pathogenic species such as ETBF, cleaves the extracellular domain of E-cadherin, reducing cell-cell adhesion and disrupting the E-cadherin/β-catenin complex. After release, β-catenin moves into the nucleus, where it triggers NF-κB-dependent inflammatory signaling and activates oncogenic Wnt targets. This inflammatory state stimulates high-level IL-6 secretion from epithelial and mucosal immune cells. IL-6 binds membrane-bound or soluble IL-6R to form a signaling complex that binds to gp130, thereby activating JAK1/2 and phosphorylating STAT3 at Tyr705. After dimerizing and translocating to the nucleus, phosphorylated STAT3 drives the transcription of genes that support angiogenesis (*VEGF*), proliferation (*c-Myc, cyclin D1*), survival (*Bcl-2, Bcl-xL*), and immunological regulation. A prolonged pro-survival and pro-proliferative transcriptional program is driven by the IL-6/STAT3 axis, which is chronically activated by biofilms‘ continuous supply of bacterial toxins and inflammatory stimuli (Huang et al., 2022; Cheng et al., 2020; Lin et al., 2020). Within these proximal colon biofilms, certain pathogens work synergistically to disrupt epithelial architecture and activate inflammatory and oncogenic cascades that accelerate tumor development. Through convergent molecular attacks on host signaling pathways, *F. nucleatum* and *enterotoxigenic Bacteroides fragilis* (ETBF) collaborate to develop a pro-carcinogenic environment within the proximal colon biofilm. F. nucleatum utilizes its *FadA* adhesin protein to bind to the extracellular domain of the host's E-cadherin molecule on epithelial cell surfaces. This interaction fractures cell-cell junctions and physically displaces the critical cytosolic protein Beta-catenin, allowing it to translocate to the nucleus where it serves as a transcriptional coactivator of Wnt target genes, promoting cell cycle progression. The ETBF bacterium also secretes the main virulence factor, the B. fragilis toxin (BFT), which is a zinc-dependent metalloprotease. Both of these events eventually lead to significant IL-6 cytokine production in the local environment. This abundant IL-6 binds to its receptor complex (gp130/IL-6R), leading to autophosphorylation of the associated JAK kinases. The active JAKs subsequently phosphorylate STAT3 at Tyr705, forming P-STAT3 homodimers that move to the nucleus. These dimers trigger the transcription of pro-survival and pro-proliferative genes, resulting in a positive feedback loop that promotes chronic inflammation and malignant transformation (Lee et al., 2022; Rubinstein et al., 2013; Chen et al., 2018). In addition to activating inflammatory and oncogenic pathways, biofilm-associated bacteria reshape the immune landscape by engaging immune-checkpoint mechanisms that suppress effective anti-tumor T-cell responses. F. nucleatum and ETBF induce molecular disruptions in proximal colon biofilms, directly contributing to immune evasion by increasing PD-L1 expression and T-cell suppression via IL-6/STAT3-dependent signaling. Persistent activation of the IL-6/STAT3 axis, triggered by bacterial virulence factors and chronic biofilm-mediated inflammation, leads to robust STAT3 phosphorylation, which increases PD-L1 transcription in epithelial, tumor, and infiltrating immune cells, thereby reducing cytotoxic T-cell activity via PD-1/PD-L1 engagement. Sustained STAT3 activity increases immunosuppressive populations, such as MDSCs and Tregs, which actively suppress CD8^+^ T-cell responses (Song et al., 2018; Xi et al., 2021). A further layer of immune-checkpoint dysregulation in biofilm-rich tumors arises from the convergence of F. nucleatum and ET (Gutierrez-Angulo et al., 2023) BF signaling on the NF-κB pathway. Both the *FadA*-*E-cadherin* association of F. nucleatum and the BFT-mediated cleavage of E-cadherin by ETBF activate NF-κB within epithelial and immune cells, resulting in a persistent inflammatory transcriptional program. The direct stimulation of the *CD274* gene, which encodes PD-L1, establishes NF-κB as a parallel driver of immune-checkpoint overexpression alongside STAT3. Persistent NF-κB activation promotes ongoing PD-L1 expression and tumor immune evasion. Furthermore, NF-κB promotes the release of pro-inflammatory cytokines and chemokines, such as IL-6 and IL-8, which disrupt the anti-tumor immune response by reducing effector T-cell activity and recruiting immunosuppressive myeloid populations. The crosstalk between biofilm-associated NF-κB signaling and STAT3 activation creates an environment that impairs T-cell-mediated tumor clearance (Rubinstein et al., 2013; Antonangeli et al., 2020; Betzler et al., 2020). In addition to its effects on epithelial signaling and inflammation, F. nucleatum deploys several specialized mechanisms to directly suppress anti-tumor immunity and reinforce immune-checkpoint activation. The outer-membrane adhesin Fap2 binds the inhibitory receptor TIGIT on NK cells and T cells, lowering their cytotoxic activity and IFN-γ production. Additionally, the CbpF protein interacts with CEACAM1 on CD4^+^ T cells, further suppressing the immune response. F. nucleatum produces metabolites such as succinic acid, which negatively impact CD8^+^ T-cell effector function. Aside from receptor-mediated inhibition, the bacteria promote intracellular signaling pathways that promote immune-checkpoint expression: ADP-heptose stimulates the ALPK1 axis, leading to PD-L1 overexpression. Activation of the cGAS-STING pathway promotes NF-κB-dependent PD-L1 transcription. STING-driven inflammation can promote immune evasion, but it may also enhance sensitivity to anti-PD-L1 treatments by increasing CD8^+^ T-cell infiltration during treatment (Galaski et al., 2021; Schöpf et al., 2025; Duizer et al., 2025; Gur et al., 2019). Beyond inflammatory and immune-modulatory effects, biofilm-associated signaling also drives epigenetic reprogramming, leading to the selective silencing of tumor-suppressor genes in affected colonic regions. Chronic inflammation caused by biofilm-associated bacteria, particularly F. nucleatum and ETBF, induces epigenetic silencing of tumor-suppressor genes (TSGs) by targeted DNA methylation rather than genetic mutation. Continuous exposure of colonic epithelial cells to pro-inflammatory cytokines such as IL-6, reactive oxygen species, and bacterial toxins creates an environment that promotes abnormal hypermethylation of CpG islands within TSG promoter regions. This promoter hypermethylation acts as a molecular “off switch,” inhibiting the transcription of essential regulators of cell cycle regulation, DNA repair, and apoptosis. These epigenetic changes aggregate over time in the biofilm-rich mucosa, locking epithelial cells into a pro-carcinogenic state and contributing to the distinctive gene-silencing patterns seen in proximal CRC (Maiuri et al., 2019; Allen et al., 2019; Li Z. et al., 2025). He IL-6/STAT3 signaling pathway is critical in sustaining these epigenetic changes in biofilm-rich regions. Chronically activated STAT3 acts not only as a transcription factor but also as an epigenetic regulator, modulating DNA methyltransferase activity and promoting promoter hypermethylation of tumor suppressor genes. Evidence from gastric cancer shows that persistent STAT3 activation can directly induce methylation-mediated silencing of genes such as *NR4A3*, and similar mechanisms are increasingly recognized in the colon, where inflammation-driven STAT3 signaling contributes to the repression of genes that normally limit tumor growth. Biofilm-associated NF-κB and IL-6/STAT3 activation led to increased DNMT expression and activity, linking microbial inflammation to host epigenomic modification (Lai et al., 2019; Thomas, 2012; Yeh et al., 2016; Lai et al., 2019). Epigenetic silencing inside biofilm-rich regions is particularly important for immune evasion because it selectively suppresses genes involved in immune recognition and response. Hypermethylation-induced inactivation of genes involved in antigen processing and presentation decreases cancer cells' accessibility to the immune system, allowing altered epithelial cells to evade cytotoxic monitoring. In the inflammatory microenvironment, immunological checkpoints such as PD-L1 are upregulated by IL-6/STAT3 and NF-κB, which together decrease epigenetic activity. Together, these activities form a dual-layered immune escape strategy in which tumor cells reduce their detectability while actively inhibiting T-cell function, thereby allowing long-term immunological tolerance and uncontrolled tumor growth (Yang and Wang, 2025; Tomasi et al., 2006; Lu et al., 2025).

Microbial genotoxicity, metabolite-driven receptor reprogramming, and biofilm-induced inflammation form an integrated pathogenic mechanism in CRC. Genotoxic colibactin-producing E. coli cause DNA double-strand breaks and micronuclei production, activating the cGAS-STING pathway and triggering NF-κB- and IRF3-mediated inflammatory signaling (Zhang et al., 2024). Increased levels of IL-6, TNF-α, and type I interferons alter epithelial metabolism, increase susceptibility to dysbiosis-derived compounds, and activate Axis II pathways, altering AhR, FXR, and TGR5 signaling from protective to pro-tumorigenic profiles (Kong et al., 2025; D'Ignazio et al., 2017; Goldiş et al., 2025). As dysbiosis progresses, reduced butyrate availability, altered indole ligands, and elevated secondary bile acids exacerbate inflammatory signaling, metabolic stress, and epigenetic remodeling (Xu et al., 2022; Lin et al., 2022). Axis III then enhances the early events: Biofilms anchored in crypts continuously secrete virulence factors, such as ETBF-derived BFT and *F. nucleatum* FadA. These factors disrupt E-cadherin, activate β-catenin and NF-κB, and promote high IL-6 production, sustaining chronic STAT3 activation (Rubinstein et al., 2013; Saravanan et al., 2024; Kushwaha et al., 2025). STAT3 and NF-κB activation boost DNMT expression and activity, leading to promoter hypermethylation and silencing of important tumor suppressors, such as MLH1, and genes involved in immune recognition, barrier integrity, and DNA repair. These epigenetic alterations, along with ROS-induced DNA damage, generate more cytosolic DNA fragments, reactivating cGAS-STING, closing a feed-forward cycle that maintains inflammation, genomic instability, immunological evasion, and receptor reprogramming (Zhang et al., 2006; Lee et al., 2012; Li W. J. et al., 2025; Zhang et al., 2005). Axes I, II, and III work together in these interconnected molecular circuits to form a single, self-reinforcing network that drives CRC initiation, development, and immune escape. This integrated loop explains how dysbiosis, metabolic rewiring, and biofilm-mediated inflammation interact to form a cohesive mechanism that promotes tumor progression and treatment resistance.

The conceptual benefit of this tri-axial framework resides in its capacity to incorporate mechanisms frequently addressed independently in the current literature. Many current models concentrate on individual microbial species, specific virulence factors, or single host signaling pathways, which can overlook the dynamic interplay among inflammation, metabolic remodeling, epithelial barrier disruption, immune evasion, and epigenetic change. In contrast, the present framework brings three interconnected axes-microbial genotoxicity, metabolite-driven receptor reprogramming, and biofilm-associated inflammatory signaling to provide a broader systems-level view of how dysbiosis influences CRC initiation, progression, and subtype heterogeneity. This approach, which emphasizes mechanistic interactions rather than individual occurrences, provides a more integrative foundation for understanding CRC biology and relating microbiome-associated processes to molecular classification, biomarker identification, and precision treatment methods.

Despite the growing body of evidence linking gut microbial dysbiosis to colorectal carcinogenesis, the current literature remains heterogeneous and, in many settings, more associative than definitively causal. Reported relationships between specific microbial taxa, metabolites, and CRC are not always consistent across studies, likely owing to differences in geographic background, diet, host genetics, medication exposure, sample type, sequencing platforms, bioinformatic pipelines, and tumor stage (Allali et al., 2017; Coker et al., 2022; Aardema et al., 2021; John Kenneth et al., 2023; Romero-Garmendia and Garcia-Etxebarria, 2022). In addition, several microbiome-related pathways appear to be highly context-dependent, with certain microbial signals or host receptors exerting protective or tumor-promoting effects depending on ligand availability, ecological composition, and disease state. Under homeostatic conditions, these pathways may support barrier integrity, immune regulation, and metabolic balance; however, in dysbiosis-associated settings they may instead amplify inflammation, oncogenic signaling, and immune suppression. This context dependency is one of the main reasons why microbiome-CRC associations are not always reproduced consistently across different studies and patient cohorts (Louis et al., 2014; Rakoff-Nahoum and Medzhitov, 2007; Donohoe et al., 2012; Gur et al., 2015). These limitations limit direct comparison across cohorts and pose significant reproducibility challenges. Therefore, metabolism evidence strongly supports a meaningful role for the microbiome in CRC-associated inflammation, metabolism, and immune modulation, caution is warranted when generalizing specific microbial signatures or mechanistic claims. Future progress will depend on better-standardized study designs, larger multi-center cohorts, and longitudinal and functional studies that can more clearly distinguish association from causation.

## 8 Implications for molecular classification and precision oncology

These three axes explain why patients with comparable histology may have markedly different genetic patterns, immunological microenvironments, and therapeutic responses. Understanding how each microbial axis influences tumor biology provides a molecular foundation for precision oncology applications in CRC. Within the tri-axial framework described above, the gut microbiota can be conceptually overlaid onto existing molecular classifications of CRC, providing a more comprehensive perspective on tumor heterogeneity.

### 8.1 Mapping microbial axes to CRC subtypes

Biofilm-associated bacteria such as *F. nucleatum* and ETBF have direct consequences for CRC molecular classification, with strong relationships between microbial signatures and specific tumor subtypes. *F. nucleatum* abundance is consistently higher in right-sided (proximal) colon cancers and correlates with key molecular features of this region, including the CpG island methylator phenotype (CIMP-high), defined by extensive promoter hypermethylation and silencing of tumor-suppressor genes. *F. nucleatum* is also significantly associated with microsatellite instability (MSI-high) tumors, indicating mismatch repair deficiency, and it frequently co-occurs with BRAF V600E mutations, defining a well-established right-sided CRC subtype. Left-sided tumors, by contrast, are more likely to be chromosomally unstable (CIN) and microsatellite stable (MSS), with weaker associations with these microbial profiles. These connections show that each microbial axis corresponds to a particular CRC subtype, suggesting that microbial signatures could improve existing molecular classifications by incorporating upstream ecological and inflammatory drivers of tumor development (Kim et al., 2018; Zlobec et al., 2012; Ono et al., 2022; Ito et al., 2015; DeDecker et al., 2021).

### 8.2 Microbial biomarkers for early detection and prognosis

Microbial signatures, particularly those containing *F. nucleatum*, are powerful noninvasive biomarkers for early detection and prognosis in CRC. Quantifying *F. nucleatum* DNA in stool samples yields high sensitivity and specificity for detecting advanced adenomas and early-stage CRC, and diagnostic performance improves further when paired with established screening tests such as the fecal immunochemical test (FIT). Circulating anti-*F. nucleatum* IgA antibodies are another potential biomarker, as they may provide serological detection. A large intratumoral burden of *F. nucleatum* is consistently associated with advanced disease stage, shorter overall survival, and decreased responsiveness to conventional chemotherapies such as 5-fluoruracil. Immune contexture also plays an important role: *F. nucleatum*-positive cancers have distinct T-cell populations, and the presence of subsets, such as FOXP3 (low) regulatory T-cell phenotypes, can improve prognosis (Roy et al., 2025; Yu et al., 2024; Shang and Liu, 2018; Zhang et al., 2018).

### 8.3 Microbiome-based therapeutic strategies

Targeting the biofilm-associated microbiota is a potential additional treatment option in CRC. In preclinical models, selective antibiotics target tumor-enriched pathogens, such as *F. nucleatum*, reduced bacterial load and suppressed cancer cell proliferation, underscoring the potential of microbe-targeted antimicrobial treatments. Fecal microbiota transplantation (FMT) is another emerging approach to restore a balanced gut ecology and re-establish homeostatic immune responses; however, its direct therapeutic benefit in CRC remains to be clinically validated. Additional strategies include the use of probiotics, such as *Lactobacillus rhamnosus*, which can boost anti-tumor immunity and potentially enhance the effects of immunotherapy, and bacteriophages, which provide highly selective elimination of pathogenic organisms like *F. nucleatum* while preserving the commensal microbiota, with the added benefit of delivering therapeutic molecules directly to the tumor microenvironment. Furthermore, small-molecule inhibitors designed to block critical bacterial virulence factors, such as the ETBF-derived BFT toxin or the *F. nucleatum* FadA adhesin, provide a precise method for disrupting microbe-driven oncogenic signaling. Together, these microbiome-focused techniques demonstrate the therapeutic potential of modifying pathogenic bacterial communities to supplement current CRC treatments (Kenneth et al., 2025; Soto Chervin and Gajewski, 2020).

### 8.4 Integration with omics and AI approaches for personalized prediction

The future of precision oncology increasingly depends on combining multi-omics data with powerful computational methods to build individualized prediction models for CRC. Multi-omics approaches that integrate gut microbiome profiling with host genomes, transcriptomics, epigenomics, and metabolomics provide a deeper understanding of the intricate microbial-host interactions that drive tumor initiation and development. Integrative analyses have revealed strong associations between Fusobacterium abundance and host genes regulating tight junction integrity, such as *Cldn7*, and oncogenic pathways, such as Wnt/β-catenin (Klf3). This demonstrates how microbial signatures map onto distinct host molecular states. AI and machine learning (ML) are critical tools for analyzing these large, multidimensional datasets. ML models can identify novel microbial and molecular biomarkers, stratify patients by risk, predict survival outcomes, and forecast responses to immunotherapy and other targeted treatments. Together, multi-omics integration and AI-driven analytics provide a powerful foundation for developing personalized diagnostic and therapeutic strategies in CRC (Karaman et al., 2025; Yang et al., 2022; Pateriya et al., 2025; Wang et al., 2025). By linking the tri-axial microbial model to molecular subtypes, diagnostics, therapeutic innovations, and AI-enabled prediction, this framework provides a unified path toward translating microbiome science into precision oncology for CRC. Table 1 illustrates a summary of the three microbial axes, associated microbes, host signaling pathways, and therapeutic implications in colorectal cancer.

**Table 1:** Summary of the tri-axial microbiota-driven framework in colorectal cancer.

Axis	Key microbes/metabolites	Host pathways activated	Major effects in CRC	Therapeutic implications
Axis I: microbial genotoxicity and cGAS–STING	*pks^+^ E. coli*, colibactin	DNA DSBs, cGAS–STING, TBK1/IRF3, NF-κB	Genomic instability, chronic inflammation	Target genotoxins, inhibit pks island, STING modulators
Axis II: metabolite-driven receptor reprogramming	Indoles (AhR ligands), SCFAs (butyrate), bile acids (DCA/LCA)	AhR, HDAC inhibition, FXR, TGR5	Epigenetic remodeling, metabolic shift, proliferation	Prebiotics/probiotics, SCFA restoration, FXR/TGR5 modulators
Axis III: biofilm-driven IL-6/STAT3 and immune evasion	*F. nucleatum, ETBF*, biofilm consortia	IL-6/STAT3, NF-κB, PD-L1, Treg/MDSC recruitment	Barrier loss, inflammation, immune escape, TSG silencing	Anti-biofilm therapies, STAT3 inhibitors, PD-L1 blockade, bacteriophages
Integrated crosstalk		Interaction of all above pathways	Self-reinforcing tumor-promoting loop	Multi target microbial + host pathway therapies

The table outlines the major microbial drivers, host signaling pathways, their roles in colorectal tumorigenesis, and potential therapeutic implications.

## 9 Conclusion

CRC is a multifactorial disease driven by complex interactions among host genetics, environmental exposures, lifestyle factors, and, most importantly, the gut microbiota. Dysbiosis is now recognized as a driver and amplifier of CRC pathogenesis through its effects on chronic inflammation, genomic instability, immunoregulation, and metabolic reprogramming. Lifestyle changes, including increased consumption of processed foods, lower dietary fiber intake, and reduced physical activity, have exacerbated microbial imbalance, contributing to the worrisome rise in early-onset CRC globally. Dysbiosis compromises the integrity of the intestinal barrier, increases permeability, and promotes persistent inflammatory signals, all of which contribute to neoplastic transformation. This review proposes a unified tri-axial mechanistic framework to consolidate the diverse microbial pathways contributing to CRC: Axis I: microbial genotoxicity and cGAS-STING-mediated innate inflammation; Axis II: microbial metabolite-driven reprogramming of host receptors such as AhR, FXR, and TGR5; and Axis III: crypt-anchored biofilm development that promotes IL-6/STAT3 activation and immune evasion. These axes work together to establish a self-perpetuating loop of DNA damage, inflammatory amplification, metabolic dysregulation, epigenetic remodeling, and immunosuppression. Pathogens such as pks^+^ E. coli, F. nucleatum, and enterotoxigenic Bacteroides fragilis use virulence factors, including colibactin, *FadA, Fap2*, and *BFT*, to activate oncogenic pathways such as NF-κB, Wnt/β-catenin, PI3K-AKT, and STAT3. Persistent activation of these pathways contributes to CIN, MSI, and CIMP characteristics in CRC and also enables tumor immune evasion via PD-L1 upregulation and cytotoxic lymphocyte suppression. Integrating these microbial pathways into well-established molecular categories reveals robust links between dysbiosis-driven axes and specific tumor subtypes. Right-sided, CIMP-high, and MSI-high malignancies are enriched for F. nucleatum and biofilm-dominated microenvironments, whereas CIN-dominant left-sided tumors are more closely associated with Axis I genotoxicity. Microbial signatures, such as F. nucleatum DNA, virulence-factor genes, and metabolite profiles, are becoming useful biomarkers for early identification, prognosis, and treatment stratification. FMT, probiotics, bacteriophages, and small-molecule inhibitors of microbial virulence pathways are intriguing additions to traditional CRC therapy. Multi-omics technologies, when integrated with AI and machine learning, have the potential to transform disease detection, risk assessment, and treatment response by incorporating microbial, metabolic, genomic, and epigenomic data into individualized prediction models. This tri-axial approach redefines CRC not just as a genetic or environmental illness but also as a complex ecosystem disturbance shaped by dynamic microbe-host interactions. A better understanding of these microbial networks opens new possibilities for precision oncology techniques, including personalized preventive, diagnostic, and therapeutic efforts that reflect the biological diversity of CRC. Collectively, these insights position the gut microbiome as a central determinant of CRC heterogeneity and highlight the transformative potential of microbiota-informed precision oncology.
